# Multiparametric magnetic resonance imaging in mucosal primary head and neck cancer: a prospective imaging biomarker study

**DOI:** 10.1186/s12885-017-3448-5

**Published:** 2017-07-10

**Authors:** Christopher N Rumley, Mark T Lee, Lois Holloway, Robba Rai, Myo Min, Dion Forstner, Allan Fowler, Gary Liney

**Affiliations:** 1 Department of Radiation Oncology, Cancer Therapy Centre, Liverpool Hospital, Liverpool, NSW Australia; 2 South Western Clinical School, School of Medicine, University of New South Wales, Sydney, NSW Australia; 3 Ingham Institute for Applied Medical Research, Liverpool, NSW Australia; 4 School of Science and Health, Western Sydney University, Parramatta, NSW Australia; 5 Centre for Medical Radiation Physics, University of Wollongong, Wollongong, NSW Australia; 6 Institute of Medical Physics, School of Physics, University of Sydney, Camperdown, NSW Australia; 7 Sunshine Coast University Hospital, Birtinya, QLD Australia

**Keywords:** Head and neck cancer, MRI, Functional imaging, Hypoxia, Predictive role, Chemoradiation, Dynamic contrast-enhanced MRI, Diffusion-weighted imaging

## Abstract

**Background:**

Radical radiotherapy, with or without concomitant chemotherapy forms the mainstay of organ preservation approaches in mucosal primary head and neck cancer. Despite technical advances in cancer imaging and radiotherapy administration, a significant proportion of patients fail to achieve a complete response to treatment. For those patients who do achieve a complete response, acute and late toxicities remain a cause of morbidity. A critical need therefore exists for imaging biomarkers which are capable of informing patient selection for both treatment intensification and de-escalation strategies.

**Methods/design:**

A prospective imaging study has been initiated, aiming to recruit patients undergoing radical radiotherapy (RT) or chemoradiotherapy (CRT) for mucosal primary head and neck cancer (MPHNC). Eligible patients are imaged using FDG-PET/CT before treatment, at the end of week 3 of treatment and 12 weeks after treatment completion according to local imaging policy. Functional MRI using diffusion weighted (DWI), blood oxygen level-dependent (BOLD) and dynamic contrast enhanced (DCE) sequences is carried out prior to, during and following treatment. Information regarding treatment outcomes will be collected, as well as physician-scored and patient-reported toxicity.

**Discussion:**

The primary objective is to determine the correlation of functional MRI sequences with tumour response as determined by FDG-PET/CT and clinical findings at 12 weeks post-treatment and with local control at 12 months post-treatment. Secondary objectives include prospective correlation of functional MRI and PET imaging with disease-free survival and overall survival, defining the optimal time points for functional MRI assessment of treatment response, and determining the sensitivity and specificity of functional MRI sequences for assessment of potential residual disease following treatment. If the study is able to successfully characterise tumours based on their functional MRI scan characteristics, this would pave the way for further studies refining treatment approaches based on prognostic and predictive imaging data.

**Trial registration:**

Australian New Zealand Clinical Trials Registry (ANZCTR): ACTRN12616000534482 (26 April 2016).

## Background

Mucosal primary head and neck cancer (MPHNC) has an estimated incidence of over 900,000 cases per year, and is associated with significant mortality, causing more than 350,000 deaths per year[1]. Organ preservation strategies involve the administration of a radical dose of radiation therapy, usually with concomitant chemotherapy, producing 5-year overall survival rates of between 30 and 50% [2]. Despite technical improvements in radiotherapy, significant toxicity may result from these treatments [3, 4].

Prognostication and treatment strategies are informed by clinical features [5] and increasingly by tumour biological features, notably preceding infection with oncogenic strains of human papilloma virus (HPV) [6, 7]. Reliable imaging biomarkers may therefore provide useful data to facilitate adaptive approaches including treatment intensification for poor responders and de-intensification for good responders [8]. Such strategies may improve the therapeutic ratio by reducing dose to organs at risk and increasing dose to areas harbouring subvolumes of more resistant tumour.

Positron-emission tomography using ^18^F–fluorodeoxyglucose (FDG-PET) is currently a standard investigation for initial staging of MPHNC. Retrospective studies have described FDG-PET’s performance in assessing disease following radical radiotherapy [9, 10] and its value in decision-making around surgical salvage has been demonstrated in a prospective trial [11]. Some limitations remain however, with the possibility of false-positive results due to benign tissue inflammation and of false-negative results due to limited spatial resolution.

Magnetic resonance imaging (MRI) has an established role in diagnosis and staging of MPHNC, and its use in radiotherapy planning continues to expand [12]. MRI has superior spatial resolution, better soft tissue definition and does not employ ionising radiation. Furthermore, functional sequences can provide additional information on tissue composition and biology which is not revealed by standard anatomical T1 and T2 weighted scans.

Diffusion-weighted imaging (DWI) characterises tissue based on movement of water within a volume. The diffusivity of water molecules depends on microstructural features of the tissue, such as cellular size and density, and may be quantified with the apparent diffusion coefficient (ADC). Its applications in the upper aerodigestive tract were previously limited by technical factors such as susceptibility artefact from air-tissue interfaces and low signal-to-noise ratio. Modern scanning techniques have been able to overcome many of these issues, and DWI measurements before, during and following RT have been investigated as a diagnostic and prognostic tool [13–18].

Dynamic contrast-enhanced (DCE) MRI provides information on abnormal tumour vasculature, by assessing the travel of gadolinium contrast agent between intravascular and extracellular extravascular space (EES) by means of sequential T1-weighted scans with high temporal resolution. With knowledge of the pre-contrast T1 tissue values, and the concentration-time course of contrast in feeding blood vessels, pharmacokinetic modelling can generate quantitative parameters for analysis of tumour perfusion [19]. These include K^trans^ (the volume transfer constant between blood plasma and EES), K_ep_ (the rate constant between EES and blood plasma) and V_e_ (the fractional volume of EES). The values of these parameters, as well as their distribution in the volume imaged, have been investigated as potential imaging biomarkers both before and after treatment for MPHNC [20–30].

Blood oxygen level–dependent (BOLD) MRI utilises local changes in magnetic susceptibility due to the presence of paramagnetic deoxyhaemoglobin. An increase in deoxyhaemoglobin shortens transverse relaxation time T2*, giving a qualitative indication of tissue hypoxia [31]. The rate of transverse relaxation R2* (1/T2*) can be measured with a multiple-echo gradient echo sequence and has been shown to correlate with tissue hypoxia as measured by polarographic electrodes and immunohistochemistry [32–34]. Pre-clinical and early clinical results suggest that R2* may be a useful biomarker for radiation response and for treatment outcomes [35–37].

Our group completed a pilot study of functional MRI and FDG-PET in a cohort of patients undergoing radical radiotherapy for MPHNC, indicating that carrying out sequential DWI and R2* studies before, during and after radiotherapy was feasible. Initial results, including correlations between imaging studies have been published, while mature data regarding clinical outcomes are awaited [18]. Following this we have initiated a prospective observational study named “MRI in MPHNC”, which aims to gather data on the utility of functional imaging sequences with DWI, DCE and BOLD MRI as biomarkers for prediction and assessment of treatment response.

## Methods

### Study design and consent

This study is a prospective single arm observational trial of patients undergoing curative intent primary radiotherapy, with or without chemotherapy, for MPHNC at Liverpool and Campbelltown Hospitals. Patients are identified and evaluated by the multidisciplinary team. Informed consent is obtained by medically trained personnel as per the trial delegation log. Administrative support, including travel funding for study scans, is provided by clinical trials unit staff.

### Hypotheses

1. Functional MRI can be used as a reliable imaging biomarker in prediction of treatment response (radiotherapy ± chemotherapy) in MPHNC
2. Functional MRI can be used as an alternative functional imaging study to FDG-PET scan in assessing response after radiotherapy ± chemotherapy
3. Functional MRI can differentiate between recurrent or residual malignant disease and post-treatment tissue changes following radiotherapy ± chemotherapy

### Objectives

#### Primary objective

To determine the correlation of pre-, during treatment and post-radiotherapy (± chemotherapy) functional MRI with tumour response at 3 months by FDG-PET scan and with local control at 12 months.

#### Secondary objectives

1. To prospectively correlate different MRI sequences and FDG-PET findings with disease-free survival (DFS) and overall survival (OS)
2. To determine the best time to perform MRI during radiotherapy treatment for assessing tumour response and local control
3. To determine the sensitivity and specificity of functional MRI sequences compared with FDG-PET in assessment of potential residual and recurrent MPHNC following definitive radiotherapy
4. To assess consistency of image registration, signal information and associated analyses over a relevant time frame in a representative subset of patients

### Endpoints

#### Primary endpoints

Correlation of functional MRI sequences with PET-CT at 3 months following treatment and local control at 12 months, including:

- Measurement of restricted water diffusion as on DWI, which can be a marker of high cellularity within a tumour, using ADC values, ΔADC and parameter maps of ADC.
- Measurement of tumour perfusion characteristics from DCE-MRI, using parameter maps and calculated values based on inflow and outflow (K^trans^, K_ep_) as well as degree of contrast enhancement of tissues (Relative Signal Intensity, RSI).
- Tumour size as defined on anatomical imaging using T2-weighted and/or T1-weighted Volume Interpolated Breathhold Examination (VIBE) Dixon MRI.

#### Secondary endpoints

Correlation of different MRI measurements with 1 and 2 year DFS and OS including:

- Determination of the ability of DWI for detecting residual or recurrent cancer
- Assessment of functional MRI in correlation with FDG-PET metabolic response at 3 months and local control at 12 months

### Subject selection

#### Inclusion criteria

- 18 years or older
- Have the ability to give informed consent
- Histologically-proven invasive mucosal primary squamous cell carcinoma of head and neck region or patients with tumours strongly suspicious for mucosal primary head and neck cancer due to clinical features AND fine needle aspiration (FNA) cytology assessment
- Primary mucosal head and neck cancer (≥T2 and/or ≥N1) AND no evidence of metastatic disease on staging PET/CT or CT (chest ± abdomen ± pelvis)
- Patient undergoing curative intent primary radiotherapy ± chemotherapy

#### Exclusion criteria

- Contraindication to MRI studies
  - Significant claustrophobia
  - Pacemaker/implantable defibrillator
  - Implanted metals e.g. Intraocular clips
  - Known allergic reaction to gadolinium (Gd)-DTPA
- Previous radiotherapy to the area to be treated
- Primary cancer surgery to the affected area
- Other malignancy within 5 years of the current diagnosis, with the exception of successfully treated basal cell or squamous cell skin carcinoma, in situ melanoma, or carcinoma in situ of the cervix

#### Early withdrawal of subjects

If a patient expresses wishes to withdraw from the trial, site staff will explain the importance of maintaining follow-up. A patient may withdraw, or be withdrawn, from the trial for the following reasons:

- Unacceptable toxicity
- Requests or requires early discontinuation for any reason
- Intercurrent illness which prevents further treatment/follow-up
- Withdrawal of consent for treatment by patient
- Develops, during the course of the study, symptoms or conditions listed in the exclusion criteria
- Investigator discretion

The investigator will also withdraw all subjects from the study if the study is terminated. Subjects are free to withdraw from the study at any time upon their request or the request of their legally acceptable representative.

Patients may re-consent if they change their mind following withdrawal, to resume participation on this trial. The patient will however remain in the trial for the purposes of follow-up and data analysis unless they specifically request otherwise.

### Radiotherapy

All participants will receive standard treatment according to locally agreed protocols [38], based on international evidence-based practice guidelines. This study does not involve a treatment intervention or additional ionising radiation exposure. Therefore, there will not be any additional radiation dose or related side effects by participating in this trial. All imaging performed with ionising radiation is part of standard clinical practice.

### Chemotherapy

All participants will be treated according to locally agreed protocols [38], based on international evidence-based practice guidelines. Therefore, there will not be any additional chemotherapy related side effects by participating in this trial.

### Imaging

#### Imaging schedule

**Fig. 1:**
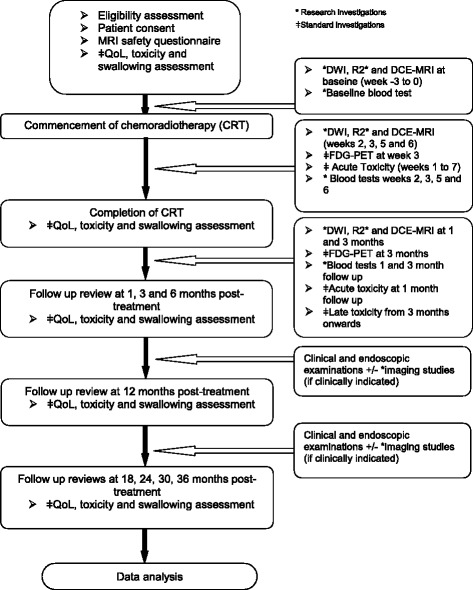
Treatment, imaging and clinical assessment schedule for MRI in MPHNC study

Functional MRI studies are carried out prior to commencing treatment, then weeks 2, 3, 5 and 6 during treatment, followed by 1 month and 3 months post-treatment (Fig. 1).

#### Imaging procedures

**Fig. 2:**
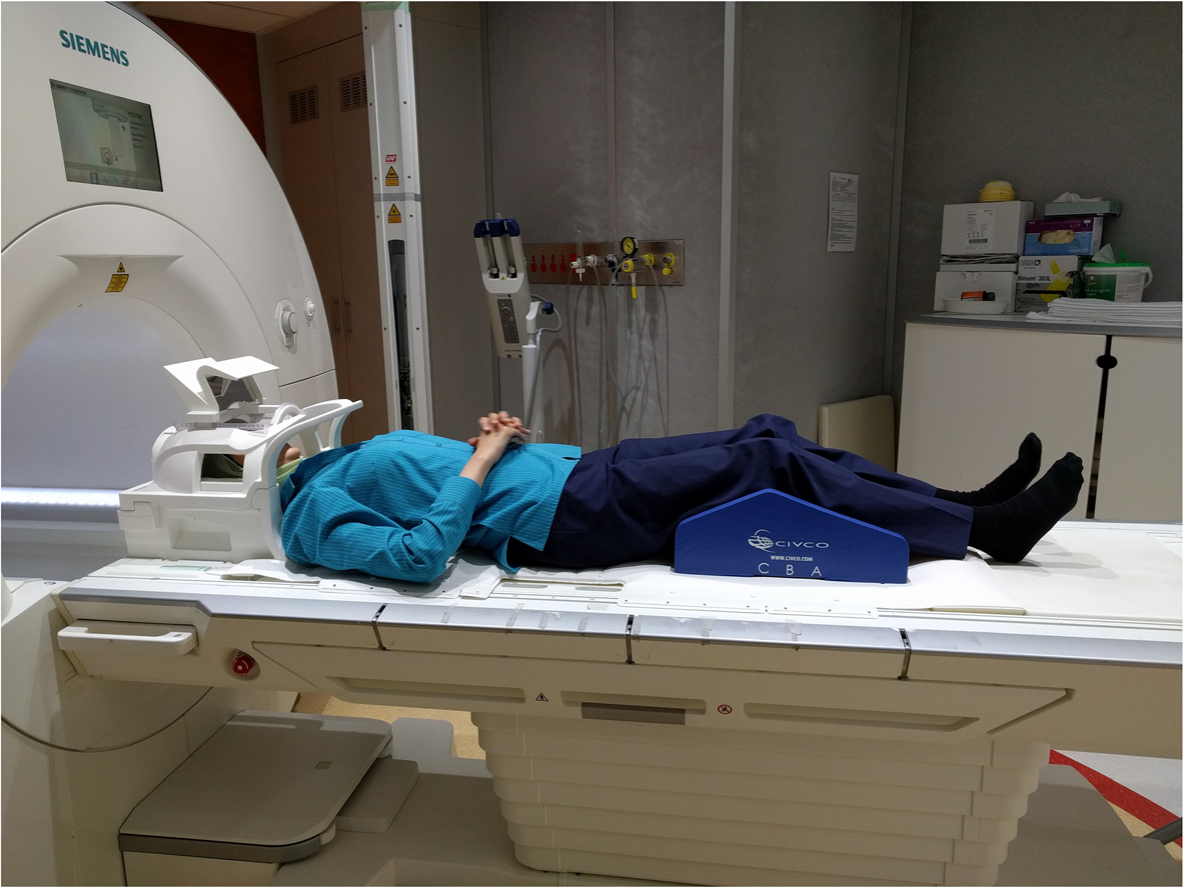
Research scan patient setup for MRI in MPHNC study

All patients scanned on a 3 T wide bore scanner (Skyra, Siemens, Erlangen, Germany) using a 20-channel head and neck coil (Fig. 2). Morphological and functional sequences are obtained in the same scan session.

##### Morphological MRI

Sagittal T2 TIRM (turbo inversion recovery magnitude).

Axial T2 TSE DIXON water-only.

Coronal T2 TSE DIXON water-only.

Axial T1 DIXON water-only, pre- and post-contrast.

##### Functional MRI

Coronal BOLD

- Gradient echo imaging with multiple echoes
- 5 mm slice thickness, 2 averages, 8 slices through region of interest
- In-plane resolution 1.7 × 1.7 mm
- TR 500 ms, TE 2.46 to 56.58 ms (12 echoes, 4.92 ms spacing)

Axial DWI

- Readout segmentation of long variable echo-trains (RESOLVE) sequence
- 3 mm slice thickness
- In-plane resolution 0.4 × 0.4 mm
- 3-scan trace, monopolar
- b = 50 s/mm^2^ with one average, b = 800 s/mm^2^ with 3 averages, calculated image at b = 1400 s/mm^2^
- Bandwidth 868 Hz/Px

Repeat coronal BOLD

- Gradient echo imaging with multiple echoes
- 5 mm slice thickness, 2 averages, 8 slices through region of interest
- In-plane resolution 1.7 × 1.7 mm
- TR 500 ms, TE 2.46 to 56.58 ms (12 echoes, 4.92 ms spacing)

DCE scans

- 2 x 3D coronal T1 VIBE (volumetric interpolated breath-hold examination) scans for T1-mapping
  - 2° and 15° flip angles
  - 3 mm slice thickness
  - In-plane resolution 1 × 1 mm
  - Acquisition time 1 m 21 s
  - Bandwidth 440 Hz/Px
- TWIST (Time-resolved angiography With Interleaved Stochastic Trajectories)
  - Slice thickness 3 mm
  - In-plane resolution 1 × 1 mm
  - TR 3.06 ms, TE 1.35 ms
  - Bandwidth 800 Hz/Px
  - i.v. gadobutrol (Gadovist, Bayer) 0.1 mL/kg, capped at a maximum of 7.5 mL; injection of contrast immediately before 4th measurement, 4 ml/s with 20 ml saline flush
  - Scan for total 60 timepoints, temporal resolution 5.64 s
  - Data acquired for 5 m 33 s

Hop on hop off sub-study scans (week −3 to 0 only)

- Patients who consent to the sub-study get up from the couch and are repositioned a few minutes later
- Axial T1, T2 and DWI scans are repeated

### Statistical plan

#### Sample size determination

Based on our previous study using week 3 FDG-PET scan in head and neck cancer patients, the following sample size calculation is made. To test a significant FDG-PET complete metabolic response (CMR) rate of 87% in the MRI-positive group versus an FDG-PET CMR rate of 51% in the MRI-negative group, the study will need to recruit 55 patients in total to reach 80% power (at 5% significance level). This number is based on an assumption that the MRI-positive group and MRI-negative group will be balanced (e.g. 50%:50% but is powered to allow up to a 40%:60% biased ratio). It is anticipated that recruitment will be completed in 24 months. Patients will be offered participation in the “hop on hop off” sub-study, with a recruitment goal of 20.

#### Assessment of MRI sequences

MRI scans will be determined as a good response (e.g. positive) or poor response (e.g. negative) based on the following definitions for endpoint analysis:

##### DWI

High ADC values will be considered a good response (positive MRI) and low ADC values a poor response (negative MRI).

##### DCE MRI

A reduction in blood inflow and outflow (e.g. K^trans^ and K_ep_) will be considered a good response (positive MRI) and either no change or an increase will be considered a poor response (negative MRI).

##### BOLD

A reduction in R2* values will be considered a good response (positive MRI) and either no change or increase in R2* values will be considered a poor response (negative MRI).

#### Definition of local control

FDG-PET response at 12 weeks will be defined as a complete metabolic response, partial metabolic response, stable metabolic disease or progressive metabolic disease based on visual assessment by the nuclear medicine physician’s report. Local control for patients will be assessed at 12 months as indicated by having no evidence of local disease on clinical (including endoscopic) examinations and imaging studies at 12 months. Tumour response will be assessed using Response Evaluation Criteria In Solid Tumors (RECIST) criteria version 1.1[39] which is the internationally recognised gold standard assessment method on the evaluation of therapeutic response in solid tumours.

#### Statistical analysis

Statistical analyses will be performed by using software SPSS and *p* < 0.05 will be considered statistically significant. Chi-square test will be used to compare the prevalence of FDG-PET CMR between MRI-positive and negative groups. Comparisons of the ADC values/maps of pre-, intra- and post-treatment DWI images will be performed by using one way analysis of variance (ANOVA) test. Comparisons of the DCE parameters such as K^trans^, K_ep_, area under the curve (AUC) at initial 60 and 90 s of pre-, intra- and post-treatment will be performed by using the Kruskal-Wallis test. To determine the sensitivity and specificity of DWI and DCE parameters for FDG-PET CMR and 12 month local control, Kappa association statistics will be used. Receiver operator characteristics (ROC) will be used to obtain an optimal threshold for individual MRI parameters.

### Data collection

General baseline demographic data will be collected. Other data collection can be defined by the 6 general categories below:

#### Quality of life

- Baseline
- Final week of chemoradiotherapy
- Follow up visits 1, 3, 6, 12, 18, 24, 30 and 36 months post treatment
- Assessed using University of Washington Quality of Life Questionnaire (UW-QOL v4)[40]

#### Blood tests

- Baseline
- Weeks 2, 3, 5, 6
- Follow up visits 1 and 3 months post-treatment
- Consist of full blood count and basic biochemistry panel including urea, creatinine, sodium, potassium and chloride

#### Toxicity

- Acute toxicities – dry mouth, dysphagia, pain, weight loss, oral mucositis, radiation dermatitis, dygeusia,
  - Baseline, weekly during treatment
  - Follow up visits 1 and 3 months post treatments
- Late toxicities – dry mouth, skin induration, osteonecrosis of jaw, voice alteration, oral mucositis, dysphagia, hearing, tracheostomy, laryngectomy, tube feeding
  - At follow up visits 3, 6, 12, 18, 24, 30 and 36 months post treatment
- Using CTCAE version 4.0
- Serious adverse events to be reported to the HREC for review

#### Swallowing assessment

- Baseline
- Final week of chemoradiotherapy
- Follow up visits 1, 3, 6, 12, 18, 24, 30 and 36 months post treatment

#### Scans

- Functional MRI scans as per Fig. 1 and *Imaging procedures* above
- As per standard of care, a PET/CT will be carried out at week 3 of chemoradiotherapy treatment and then at 3 months following completion of chemoradiotherapy treatment

#### Treatment data

- The dose and duration of treatment will be recorded.

### Data handling and record keeping

All imaging datasets will be de-identified and stored in a secure research drive at the Cancer Therapy Centre, Liverpool Hospital after recruiting patients to the study. Contours will be performed by radiation oncologists/fellows and saved on the research drive. Datasets will be imported into MATLAB (MathWorks Inc. Massachusetts, United States) and similar mathematical analysis programs for data-analysis. Data analysis will be in line with study aims.

The investigator site file and patient study file will be kept at the Ingham Institute Liverpool and will be accessible only by clinical trials staff.

#### Confidentiality

Subject confidentiality is strictly held in trust by the participating investigators, research staff, the sponsoring institution and their agents. The study documentation, data and all other information generated will be held in strict confidence. No information concerning the study or the data will be released to any unauthorized third party, without prior written approval of the HREC and the principal investigator. Clinical information will not be released without written permission of the subject, except as necessary for monitoring by Human Research Ethics Committee (HREC) or regulatory agencies.

#### Records retention

It is the investigators’ responsibility to retain study essential documents for at least 15 years after the completion of this clinical trial. All information will be stored in the Radiation Oncology research office located at the Ingham Institute, Liverpool Hospital, either on a password protected computer or in files kept in a locked room. Access to this information will be limited to the principal investigator, research assistants and statistician as authorized by the delegation log.

### Timelines/milestones

The study started recruitment after ethics approval was granted. A sample size of 55 has been selected for this study for the reasons discussed above. Initial projections suggested this could be achieved within 24 months, however this timeframe will be reviewed as recruitment progresses.

### Publication and dissemination

The results of the study will be submitted for publication in peer-reviewed journals and for presentation at scientific meetings. Participants in the study will be notified of study results in writing.

## Discussion

In recent years intensity-modulated RT, along with advanced diagnostic and on-treatment imaging technologies, have improved the therapeutic ratio in head and neck cancer [41]. The enhanced ability to dose-paint and the recognition of heterogeneity within tumours gives rise to the promise of functional image-guided RT (fIGRT), where subvolumes may be treated to differing doses according to imaging-derived parameters. Longitudinal diffusion-weighted imaging has already been shown to be feasible in MRI-guided cobalt radiotherapy systems [42], suggesting that the addition of functional as well as anatomical imaging may serve to improve outcomes with these systems, as well as in the MRI-guided linear accelerators in development.

Our protocol builds on our earlier work which established the feasibility of serial functional MRI during a course of treatment for MPHNC. Refinements to the imaging protocol include the addition of DCE sequences, which may be expected to add further value [43], and the expansion of the BOLD sequence to acquire multiple slices across a volume, an imaging technique which may be more reliable than BOLD imaging carried out on single slices [44]. The additional information gained from the enhanced suite of imaging sequences in the current study will help to define which sequences, at which time points, can be used to adapt and personalise radiotherapy treatment. This information will then feed into the design of interventional studies of adaptive RT.

## Abbreviations

ADC: Apparent diffusion coefficient
AUC: Area under the curve
BOLD: Blood oxygen level-dependent
CMR: Complete metabolic response
CRT: Chemoradiotherapy
CT: Computerised tomography
CTCAE: Common terminology criteria for adverse events
DCE: Dynamic contrast-enhanced
DFS: Disease free survival
DWI: Diffusion-weighted imaging
EES: Extracellular extravascular space
FDG-PET: Fluoro-deoxy glucose positron emission tomography
fIGRT: Functional image-guided radiotherapy
FNA: Fine needle aspiration
Gd: Gadolinium
HPV: Human papilloma virus
HREC: Human research ethics committee
IV: Intravenous
K_ep_: Rate constant between extracellular extravascular space (EES) and blood plasma
K^trans^: Volume transfer constant between blood plasma and extracellular extravascular space (EES)
MPHNC: Mucosal primary head and neck cancer
MRI: Magnetic resonance imaging
OS: Overall survival
PET: Positron emission tomography
QoL: Quality of life
R2*: Relaxation rate for T2* (1/T2*)
RESOLVE: Readout segmentation of long variable echo-trains
ROC: Receiver operator characteristic
RSI: Relative signal intensity
RT: Radiotherapy
TIRM: Turbo inversion recovery magnitude
TSE: Turbo spin echo
TWIST: Time-resolved angiography with interleaved stochastic trajectories
V_e_: Fractional volume of extracellular extravascular space (EES)
VIBE: Volume interpolated breath-hold examination
